# Evaluation of Quantitative Differences of Classic Methods, Fungal Biomarkers, and T2Candida for Assessing the Central Venous Catheter Origin of Candidemia

**DOI:** 10.1093/ofid/ofag411

**Published:** 2026-07-13

**Authors:** Ana Soriano-Martín, Antonio Vena, María Jesús Pérez-Granda, Alessandro Limongelli, Almudena Burillo, Pilar Escribano, Jesús Guinea, Malgorzata Mikulska, Daniele Roberto Giacobbe, Marina Machado, Roberto Alonso, Anna Marchese, Paola Morici, Matteo Bassetti, Emilio Bouza, Patricia Muñoz

**Affiliations:** Clinical Microbiology and Infectious Diseases Department, Hospital General Universitario Gregorio Marañón, Madrid, Spain; Instituto de Investigación Sanitaria Hospital Gregorio Marañón (IiSGM), Madrid, Spain; CIBER Enfermedades Respiratorias-CIBERES (CIBERES CB06/06/0058), Madrid, Spain; Infectious Diseases Unit, Policlinico San Martino Hospital—IRCCS, Genoa, Italy; Department of Health Sciences (DISSAL), University of Genoa, Genoa, Italy; Clinical Microbiology and Infectious Diseases Department, Hospital General Universitario Gregorio Marañón, Madrid, Spain; Instituto de Investigación Sanitaria Hospital Gregorio Marañón (IiSGM), Madrid, Spain; CIBER Enfermedades Respiratorias-CIBERES (CIBERES CB06/06/0058), Madrid, Spain; Department of Nursing, School of Nursing, Physiotherapy and Podiatry, Universidad Complutense de Madrid, Madrid, Spain; Infectious Diseases Unit, Policlinico San Martino Hospital—IRCCS, Genoa, Italy; Department of Health Sciences (DISSAL), University of Genoa, Genoa, Italy; Clinical Microbiology and Infectious Diseases Department, Hospital General Universitario Gregorio Marañón, Madrid, Spain; Instituto de Investigación Sanitaria Hospital Gregorio Marañón (IiSGM), Madrid, Spain; CIBER Enfermedades Respiratorias-CIBERES (CIBERES CB06/06/0058), Madrid, Spain; Medicine Department, School of Medicine, Universidad Complutense de Madrid, Madrid, Spain; Clinical Microbiology and Infectious Diseases Department, Hospital General Universitario Gregorio Marañón, Madrid, Spain; Instituto de Investigación Sanitaria Hospital Gregorio Marañón (IiSGM), Madrid, Spain; CIBER Enfermedades Respiratorias-CIBERES (CIBERES CB06/06/0058), Madrid, Spain; Clinical Microbiology and Infectious Diseases Department, Hospital General Universitario Gregorio Marañón, Madrid, Spain; Instituto de Investigación Sanitaria Hospital Gregorio Marañón (IiSGM), Madrid, Spain; CIBER Enfermedades Respiratorias-CIBERES (CIBERES CB06/06/0058), Madrid, Spain; Infectious Diseases Unit, Policlinico San Martino Hospital—IRCCS, Genoa, Italy; Department of Health Sciences (DISSAL), University of Genoa, Genoa, Italy; Infectious Diseases Unit, Policlinico San Martino Hospital—IRCCS, Genoa, Italy; Department of Health Sciences (DISSAL), University of Genoa, Genoa, Italy; Clinical Microbiology and Infectious Diseases Department, Hospital General Universitario Gregorio Marañón, Madrid, Spain; Instituto de Investigación Sanitaria Hospital Gregorio Marañón (IiSGM), Madrid, Spain; Universidad de Alcalá, Escuela de Doctorado, Alcalá de Henares, Spain; Clinical Microbiology and Infectious Diseases Department, Hospital General Universitario Gregorio Marañón, Madrid, Spain; Instituto de Investigación Sanitaria Hospital Gregorio Marañón (IiSGM), Madrid, Spain; CIBER Enfermedades Respiratorias-CIBERES (CIBERES CB06/06/0058), Madrid, Spain; Medicine Department, School of Medicine, Universidad Complutense de Madrid, Madrid, Spain; Department of Surgical Sciences and Integrated Diagnostics (DISC), University of Genoa, Genoa, Italy; Microbiology Unit, IRCCS Ospedale Policlinico San Martino, Genoa, Italy; Department of Surgical Sciences and Integrated Diagnostics (DISC), University of Genoa, Genoa, Italy; Microbiology Unit, IRCCS Ospedale Policlinico San Martino, Genoa, Italy; Infectious Diseases Unit, Policlinico San Martino Hospital—IRCCS, Genoa, Italy; Department of Health Sciences (DISSAL), University of Genoa, Genoa, Italy; Clinical Microbiology and Infectious Diseases Department, Hospital General Universitario Gregorio Marañón, Madrid, Spain; Instituto de Investigación Sanitaria Hospital Gregorio Marañón (IiSGM), Madrid, Spain; CIBER Enfermedades Respiratorias-CIBERES (CIBERES CB06/06/0058), Madrid, Spain; Medicine Department, School of Medicine, Universidad Complutense de Madrid, Madrid, Spain; Clinical Microbiology and Infectious Diseases Department, Hospital General Universitario Gregorio Marañón, Madrid, Spain; Instituto de Investigación Sanitaria Hospital Gregorio Marañón (IiSGM), Madrid, Spain; CIBER Enfermedades Respiratorias-CIBERES (CIBERES CB06/06/0058), Madrid, Spain; Medicine Department, School of Medicine, Universidad Complutense de Madrid, Madrid, Spain

**Keywords:** (1,3)-β-D-glucan, candidemia, catheter-related candidemia, diagnosis, differentials

## Abstract

**Background:**

This study was conducted to investigate the diagnostic value of fungal biomarker differentials and T2Candida polymerase chain reaction (PCR) from catheter and peripheral blood samples and to re-evaluate classical conservative methods, such as differential time to positivity of blood cultures (BCs), for the early identification of catheter-related candidemia (CRC) before catheter removal.

**Methods:**

This prospective study was conducted (March 2023–April 2025) in 2 tertiary hospitals in Spain and Italy. Adults (aged ≥18 years) with candidemia and a central venous catheter in place at diagnosis were included, provided catheter removal occurred within 48 hours of index BC positivity. CRC was defined in patients with candidemia who showed 1 or 2 of the following criteria in the catheter tip: (1) ≥ 15 CFU/plate (Maki technique) or ≥ 1000 CFU/mL (sonication) and (2) any *Candida* growth on the catheter tip. Blood samples were collected from peripheral veins and catheter lumens to compare the differentials of 1,3-β-D-glucan (BDG), *Candida albicans* germ tube antibodies, mannan antigen, antimannan antibody, and T2Candida PCR results. Diagnostic metrics were calculated for each test.

**Results:**

Of 179 episodes of candidemia reviewed, 28 (15.6%) met the inclusion criteria (13 in Spain, 15 in Italy). CRC was confirmed in 11 patients (39.3%) using criterion 1 and in 15 patients (53.6%) using criterion 2. BDG differentials (any difference) demonstrated the highest diagnostic accuracy as follows: sensitivity 81.82%, specificity 82.35%, positive predictive value 75.00%, negative predictive value 87.50%, and overall accuracy 82.14% (criterion 1). However, stricter BDG thresholds (≥20 or ≥30 pg/mL) and the application of criterion 2 reduced the sensitivity. Other biomarkers and T2Candida PCR demonstrated low sensitivity (0%–40%) and variable specificity (25%–100%), with overall accuracies of <64%. Differential time to positivity of BCs yielded a sensitivity of 72.73% and a specificity of 52.94%, with limited accuracy.

**Conclusions:**

None of the investigated methods achieved sufficient accuracy to diagnose CRC without catheter removal. Despite the limited sample size, this study emphasizes the limitations of current diagnostic approaches and the need for novel tools for reliable noninvasive identification of CRC.

## Graphical Abstract

**Graphical Abstract:**
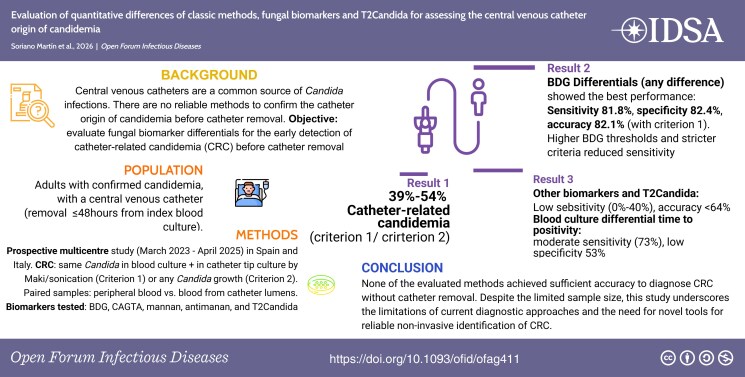
This graphical abstract is also available at Tidbit: https://tidbitapp.io/tidbits/evaluation-of-fungal-biomarkers-and-t2candida-for-the-diagnosis-of-central-venous-catheter-related-candidemia-949a8a3a-35f1-4f7f-b199-5781c3057901?utm_campaign=tidbitlinkshare&amp;utm_source=IO

Candidemia represents one of the most serious and frequent invasive fungal infections in the hospital setting with high morbidity, mortality and associated costs [1–3].

Although central venous catheters (CVCs) are considered major sources for candidemia, it is not possible to determine exactly and in an early stage which episodes of candidemia originate in these intravascular devices, because of the difficulty to confirm whether a catheter is the source of candidemia before removing it (“conservative”) and examining the removed tip [4–7].

The results of “conservative” methods to establish the catheter as the source of candidemia before its removal, such as time differential blood cultures (BCs) and other procedures, demonstrate high variability and limited validity in the case of yeast infections and cannot be extrapolated from those obtained in situations of bacteremia [7–10].

Currently, *Candida* fungal biomarkers include 1,3-β-D-glucan (BDG), *Candida albicans* germ tube antibodies (CAGTA), mannan, antimannan, and molecular techniques such as T2Candida [11, 12]. These biomarkers have been investigated as tools for the early diagnosis of candidemia and have demonstrated clinical value, particularly when used in combination, improving sensitivity and contributing to early therapeutic decisions and prognostic stratification [13–15]. Moreover, some studies suggest that catheter-related candidemia is more frequently associated with negative results on these tests [13, 14, 16]. Nevertheless, we have not found information on their ability to confirm the origin of candidemia in the catheter.

Therefore, a universal removal and replacement of CVCs is currently often adopted in cases of candidemia. However, in a high proportion of cases, the removed catheter tips do not show the presence of *Candida* [17, 18]. Due to these limitations, we consider it necessary to develop innovative strategies that allow reliable identification of catheter-related candidemia (CRC) cases without the need for catheter removal.

Hence, this prospective study was conducted to re-evaluate both classical conservative procedures and explore the use of fungal biomarker differentials and T2Candida polymerase chain reaction (PCR) obtained from catheter and peripheral blood with the aim of early detection or exclusion of CRC.

## METHODS

### Study Design, Population, and Setting

This study was conducted in 2 public hospitals, the Hospital General Universitario Gregorio Marañón in Madrid, Spain (hospital 1), which is a tertiary university hospital with 1236 beds serving an area of ≈850 000 inhabitants, and the Hospital Policinico San Martino in Genoa, Italy (hospital 2), which is a tertiary hospital with 1194 beds serving an area of ≈400 000 inhabitants.

The primary objective of this study was to investigate the efficacy of differential time to positivity of BCs and of the comparison of the ratio of fungal biomarkers—quantitative or qualitative, depending on the assay, including molecular techniques—between catheter and peripheral blood samples, as potential markers for establishing CRC diagnosis before catheter removal.

**Figure 1.**
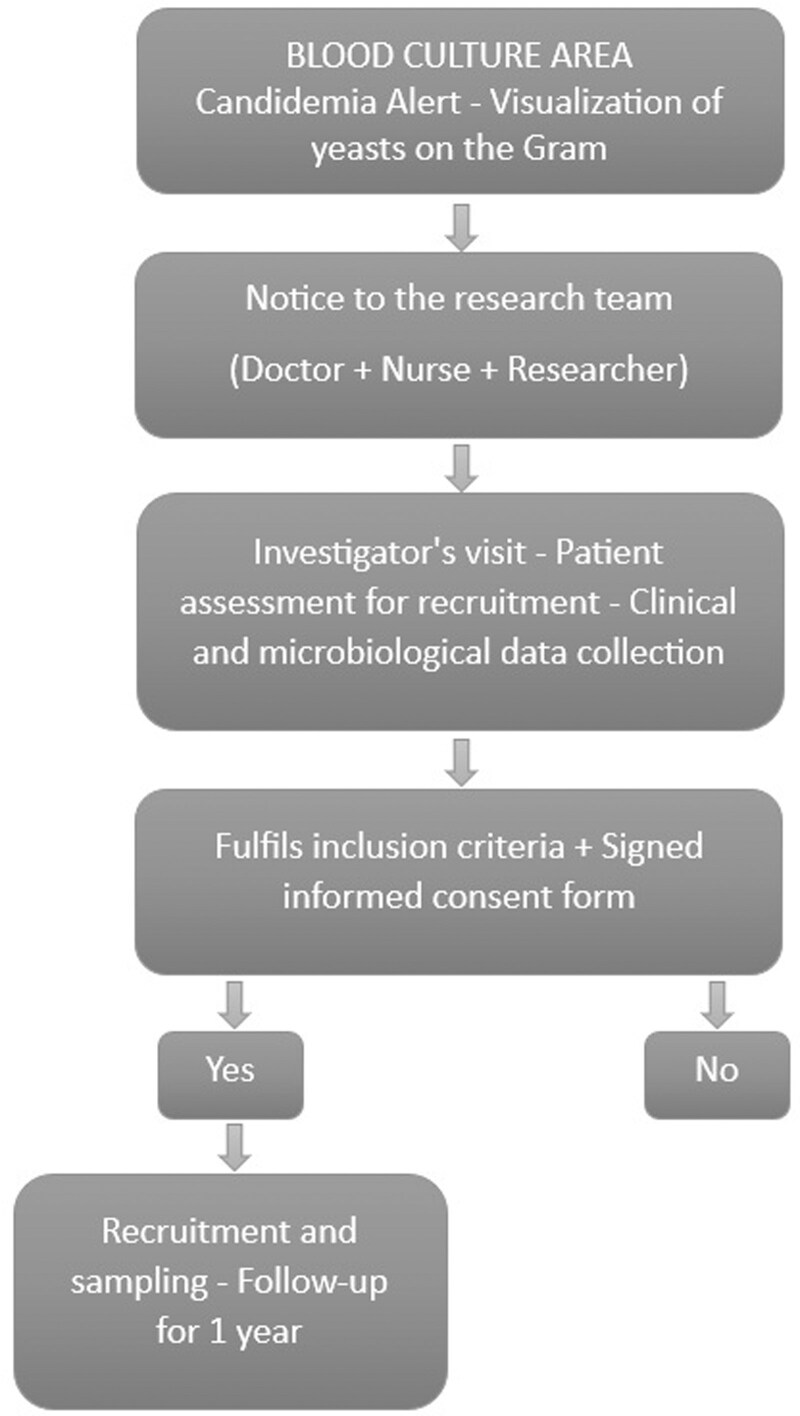
Flowchart of the recruitment procedure for patients with an episode of candidemia.

All episodes of candidemia at both institutions were prospectively examined from March 2023 to April 2025. Inclusion criteria were adult patients (aged >18 years) with candidemia confirmed by peripheral BCs, in whom at least 1 CVC remained in situ (placed before the index Bc) at the time of the study visit. Another requirement was that the catheter could be removed within 48 hours after index Bc positivity. Patients had to sign an informed consent form. Those who did not comply with the abovementioned requirements were excluded. The clinical course of the enrolled patients was followed up for 1 year after the candidemia episode. Figure 1 depicts the procedure for recruiting patients with episodes of candidemia.

### Data Collection

The data collection protocol included epidemiological, clinical, and microbiological variables such as demographic information, primary comorbidities, hospitalization unit, risk factors for candidemia, treatment and clinical course of all patients, and CVCs information. Mortality was evaluated at 30 and 60 days, with follow-up at 1 year. Information was obtained through the electronic Health Care Information System and the registry system of the microbiology department. Moreover, a digital database was generated to collect all this information anonymously.

### Microbiological Procedures

#### Identification of *Candida* spp

The processing and identification of all samples with *Candida* spp. were performed routinely according to standard guidelines and procedures, based on the clinical microbiology procedures of the Spanish Society of Infectious Diseases and Clinical Microbiology (SEIMC) [8, 19, 20].

Positive index BCs consisted of aerobic and anaerobic Bc sets with blood volumes of 5–10 mL. Bottles containing the samples were incubated at 35°C for 5 days in a BACTEC™ FX (Becton-Dickinson, Franklin Lakes, NJ,  USA) until a result was obtained.

Identification of *Candida* species was performed by subculturing followed by matrix-assisted laser desorption/ionization time-of-flight mass spectrometry (MALDI-TOF MS), using a MALDI Biotyper Smart® mass spectrometer (Bruker Daltonik, Bremen, Germany), and the result was confirmed via molecular identification by amplification and sequencing of the internal transcribed spacer 1 (ITS1) and ITS2 regions from the ribosomal DNA [21].

### Assays Performed After Signing the Informed Consent

For the investigation, blood samples were collected from each recruited patient, from both peripheral blood and each catheter lumen. All samples were subsequently distributed for BCs (5 mL per bottle, aerobic/anaerobic), T2Candida (4 mL), and serological determinations (3 mL). Samples that could not be processed on the same day or the next day after collection were frozen at −80°C.

BCs were processed as previously described and considered significant when the time difference between the catheter and peripheral line BCs was ≥2 hours. CVCs were removed within 48 hours after the index Bc was positive for *Candida* and cultured on Columbia agar plates supplemented with 5% sheep blood, using 2 methods. First, using the Maki technique, the catheter tip was rolled 3 to 4 times over the surface of a blood agar plate [22]. Next, the CVCs were also cultured using the modified sonication technique, wherein the CVC was fragmented into small portions and placed in 1 mL of sterile saline solution, vortexed, exposed to ultrasound for 1 minute at 35 000/55 000 Hz and 125 W, and vortexed again for a few seconds [23]. Subsequently, 100 μL of the liquid was quantitatively seeded, and all plates were incubated at 35°C for 48 hours. Finally, colony counts were performed.

### Serological Detection of Biomarkers

Serum BDG was detected using the Wako β-glucan diagnostic test (Fujifilm Wako Pure Chemical Corporation, Osaka, Japan) according to the manufacturer's instructions. A cutoff point of ≥ 7 pg/mL was considered positive according to the current Ce-marked manufacturer recommendations used in our region [24–26]. For detecting CAGTA (Vircell Microbiologists S.L., Granada, Spain), a titer of ≥1/160 was considered positive. For detecting mannan and antimannan, the Platelia Candida Ag Plus and Platelia Candida Ab Plus assays were used on the automated EVOLIS™ Twin Plus system (Bio-Rad, Marnes-la-Coquette, France). The manufacturer-defined cutoffs were as follows: mannan, positive ≥125 pg/mL, indeterminate 62.5–125 pg/mL, and negative <62.5 pg/mL; antimannan, positive ≥10 AU/mL, indeterminate 5–10 AU/mL, and negative <5 AU/mL.

The results of the BDG assay were analyzed considering the quantitative differences between CVC and peripheral blood samples, including any positive differential (ie, CVC BDG > peripheral BDG), as well as differences of ≥20 and ≥30 pg/mL. The results of the CAGTA assay were evaluated qualitatively, whereas mannan and antimannan determinations were analyzed both qualitatively and quantitatively.

### Molecular Techniques

The T2Candida PCR (T2MR technology, T2 Biosystems, Lexington, MA, USA) was performed on EDTA whole blood obtained from a peripheral vein and from each of the catheter lumens, according to the automated protocol described by Mylonakis et al [27]. This technique detects the major species causing invasive candidiasis, which are grouped according to their resistance profiles as follows: *C. albicans*/*C. tropicalis*, *C. krusei* (*Pichia kudriavzevii*)/*C. glabrata* (*Nakaseomyces glabrata*), and *C. parapsilosis*. In the event of invalid internal control, no positive signals were detected, and an “invalid” result was issued, indicating the possible presence of inhibitors that interfere with *Candida* detection. The results of the T2Candida test were evaluated qualitatively.

### Definitions

- An episode of candidemia was defined as at least 1 Bc obtained from peripheral veins positive for any *Candida* species [28, 29]. Patients with positive BCs obtained exclusively from catheter samples were excluded from the study.
- Persistent candidemia was defined as one or more positive control BCs obtained ≥5 days after the index BCs in patients receiving antifungal treatment [30].
- Microbiologically confirmed CRC was defined when the same *Candida* species was isolated from one or more BCs obtained from both peripheral veins and the catheter tip. For evaluating *Candida* colonization at the CVC tip, the following 2 situations were considered:
  1. Criterion 1: Semiquantitative catheter tip culture with a growth of ≥15 colony-forming units (CFU)/plate using the Maki technique or ≥1000 CFU/mL using the sonication technique [31, 32].
  2. Criterion 2: Culture of the catheter tip with any amount of *Candida* spp. isolated using the Maki technique and/or by sonication.
- The diagnosis of intra-abdominal-related candidemia required positive cultures of *Candida* spp. from intra-abdominal fluid obtained during surgery or needle aspiration, together with peritonitis or abdominal abscess.
- The urinary origin of candidemia was established when the same *Candida* spp. were isolated in urine cultures in the presence of predisposing urological diseases or conditions (eg, urinary tract manipulation or obstruction).
- Primary candidemia was defined as candidemia without a clear focus of infection (considering abdominal, urinary, or catheter-associated infection as potential origins) and without demonstrable microbiological evidence.
- Differential methods are defined as those in which the results of a given parameter are compared between blood samples obtained through the catheter lumens and those obtained from peripheral veins. Differentials are considered positive when the results from catheter blood are positive or quantitatively higher than those from peripheral blood, which are negative or quantitatively lower.

### Statistical Analysis

Statistical analyses were conducted using IBM SPSS Statistics, V26.0 (Armonk, NY, USA). Normality of continuous variables was evaluated using the Kolmogorov–Smirnov test. Nonparametric tests were applied for some variables that did not have a normal distribution. Categorical variables are expressed as frequencies and percentages, and continuous variables are represented as medians and interquartile ranges (IQRs). For group comparisons, the Mann–Whitney *U* test was used for continuous variables, and the χ^2^ test or Fisher's exact test (when at least one of the expected frequencies in a 2 × 2 table was <5) was applied for categorical variables.

Sensitivity, specificity, positive predictive value (PPV), negative predictive value (NPV), and accuracy were calculated for determining the performance of the diagnostic tests. Considering the nature of the data and the sample size, the binomial distribution was used to estimate proportions, and 95% confidence intervals were estimated using Wilson's method, due to its robustness in estimating intervals for proportions, especially in the context of small samples.

### Ethical Statement

This study was approved by the Ethics Committee for Research with Medicines of the Hospital General Universitario Gregorio Marañón and by the Regional Ethics Committee of the Hospital Policlinico San Martino (Number: MICRO.HGUGM.2021–030) on 2 November 2022, and 28 September 2023, respectively.

## RESULTS

**Figure 2.**
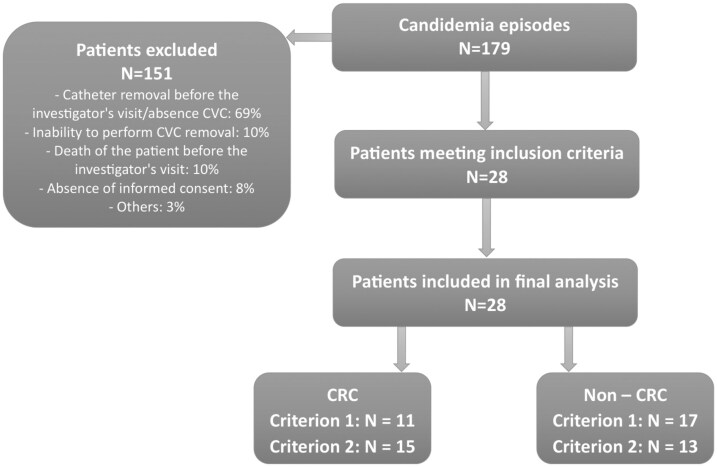
Flowchart of patient selection and inclusion in the study. CRC, catheter-related candidemia. Criterion 1: semiquantitative catheter tip culture with a growth of ≥15 colony-forming units (CFU)/plate using the Maki technique or ≥1000 CFU/mL by sonication. Criterion 2: Culture of the catheter tip with any amount of *Candida* spp. isolated using the Maki technique and/or by sonication.

During the study period, 179 episodes of candidemia were reviewed, including 74 at hospital 1 and 105 at hospital 2. Finally, 28 patients (15.6%; 13 in hospital 1 and 15 in hospital 2) who fulfilled the enrollment criteria could be recruited for the study (Figure 2). The primary reasons for patient exclusion, in the order of the highest to lowest frequency, were catheter removal before the investigator's visit, inability to perform CVC removal within a short timeframe after the study visit, death of the patient before the investigator's visit, and the absence of informed consent.

### Clinical Characteristics and Microbiological Findings of the 28 Patients Recruited

Table 1 shows the major clinical characteristics of the 28 patients included in this study and classified according to criterion 1. The most frequent comorbidities were solid tumors (35.7%) and cardiovascular disease (28.6%). At the time of diagnosis, most patients were hospitalized in medical wards (67.9%), and all candidemia cases were nosocomial. The peripherally inserted central catheter (PICC) was the most common type of CVC.

**Table 1.** Clinical Characteristics, Risk Factors, Treatment, and Outcome of the 28 Patients With Candidemia Included in This Study

Variables Studied	Total Patients N = 28 (%)	CRC N = 11 (39.3)	No CRC N = 17 (60.7)	*P* Value
Age—median years (IQR)	65.5 (55.3–79.5)	60 (55–74)	68 (55–82)	.510
Sex—male	16 (57.1)	6 (54.5)	10 (58.8)	.823
Charlson comorbidity index–median (IQR) by age	5 (4–7)	4 (4–8)	6 (4–7)	.667
Comorbidity				
Solid tumor	10 (35.7)	5 (45.5)	5 (29.4)	.387
Cardiovascular	8 (28.6)	2 (18.2)	6 (35.3)	.419
Diabetes mellitus	6 (21.4)	3 (27.3)	3 (17.6)	.653
Neurologic disease	5 (17.9)	0 (0.0)	5 (29.4)	.125
Hematologic malignancy	4 (14.3)	1 (9.1)	3 (17.6)	1.000
Gastrointestinal disease	3 (10.7)	2 (18.2)	1 (5.9)	.543
Chronic kidney disease	3 (10.7)	3 (27.3)	0 (0.0)	**.050**
Liver disease	3 (10.7)	0 (0.0)	3 (17.6)	.258
HIV	1 (3.6)	1 (9.1)	0 (0.0)	.393
Pulmonary disease	0 (0.0)	0 (0.0)	0 (0.0)	-
Hospital setting at candidemia diagnosis				
Medical ward	19 (67.9)	9 (81.8)	10 (58.8)	.203
Surgical Ward	7 (25.0)	2 (18.2)	5 (29.4)	.668
Intensive care unit	2 (7.1)	0 (0.0)	2 (11.8)	.505
Pitt score-median (IQR)	0.00 (0.00–1.75)	0.00 (0–1)	0.00 (0–2.5)	.681
Quick SOFA score—median (IQR)	0.50 (0.00–1.00)	0.50 (0.00–1.00)	1.00 (0.00–1.00)	.605
Risk factors for candidemia				
Broad-spectrum antibiotics	25 (89.3)	10 (90.9)	15 (88.2)	1.000
CVC in the past 3 m	23 (82.1)	10 (90.9)	13 (76.5)	.619
Total parenteral nutrition	18 (64.3)	6 (54.5)	12 (70.6)	.387
Previous surgery	9 (32.1)	2 (18.2)	7 (41.2)	.203
Corticosteroid therapy	9 (32.1)	4 (36.4)	5 (29.4)	.700
Previous antifungal therapy	8 (28.6)	2 (18.2)	6 (35.3)	.419
Immunosuppressive therapy	7 (25.0)	3 (27.3)	4 (23.5)	1.000
Colonization in the past 1 m	7 (25.0)	1 (9.1)	6 (35.3)	.191
Chemotherapy	7 (25.0)	5 (45.5)	2 (11.8)	.076
Neurological disease	5 (17.9)	0 (0.0)	5 (29.4)	.125
Abdominal surgery	4 (14.3)	0 (0.0)	4 (23.5)	.132
Neutropenia	4 (14.3)	1 (9.1)	3 (17.6)	1.000
Cardiac surgery	3 (10.7)	1 (9.1)	2 (11.8)	1.000
Central venous catheters				
Number of CVCs at the time of the positive blood culture–median (IQR)	1 (1.0–1.8)	1 (1.0–1.0)	1 (1.0–2.0)	.576
Number of CVCs at the time of the investigator's visit–median (IQR)	1 (1.0–2.0)	1 (1.0–2.0)	1 (1.0–2.0)	.493
Days with catheter–mean (IQR)	24 (11–46)	27 (13–81)	22 (10–39)	.268
Location				
Arm	15 (55.6)	5 (45.5)	10 (58.8)	.488
Jugular	7 (25.0)	3 (27.3)	4 (23.25)	1.000
Subclavian	4 (14.3)	1 (9.1)	3 (17.16)	1.000
Femoral	1 (3.6)	1 (9.1)	0 (0.0)	.393
PICC	16 (57.1)	5 (45.5)	11 (64.7)	.315
Central	11 (39.3)	5 (45.5)	6 (35.3)	.591
Hickman	1 (3.6)	1 (9.1)	0 (0.0)	.393
Microbiological origin of candidemia				
Primary	16 (57.1)	0 (0.0)	16 (94.1)	**<.001**
Catheter-related	11 (39.3)	11 (100.0)	0 (0.0)	**<.001**
Abdominal	1 (3.6)	0 (0.0)	1 (5.9)	1.000
Urinary	0 (0.0)	0 (0.0)	0 (0.0)	-
*Candida* spp.				
*Candida albicans*	12 (42.9)	6 (54.5)	6 (35.3)	.315
*Candida parapsilosis*	7 (25.0)	1 (9.1)	6 (35.3)	.119
*Candida glabrata (Nakaseomyces glabrata)*	4 (14.3)	2 (18.2)	2 (11.8)	1.000
*Candida krusei (Pichia kudriavzevii)*	2 (7.1)	0 (0.0)	2 (11.8)	.505
*Candida tropicalis*	1 (3.6)	0 (0.0)	1 (5.9)	1.000
*Candida lusitaniae (Clavispora lusitaniae)*	1 (3.6)	1 (9.1)	0 (0.0)	.393
*Candida auris*	1 (3.6)	1 (9.1)	0 (0.0)	.393
Days of hospitalization until the candidemia episode–median (IQR)	17.5 (6.3–36.5)	21 (6–30)	28 (6.5–43)	.588
Received antifungal therapy	28 (100)	11 (100)	17 (100)	-
First-line antifungal agent				
Echinocandin	19 (67.9)	7 (63.6)	12 (70.6)	.700
Fluconazole	7 (25.0)	3 (27.3)	4 (23.5)	1.000
Liposomal amphotericin B	2 (7.1)	1 (9.1)	1 (5.9)	1.000
Days of antifungal treatment				
Mean (DS)	17.2 (10.7)	13.8 (4.6)	19.5 (13.04)	.345
Median (IQR)	16 (12–18)	15 (12–18)	16.5 (11.8–23.8)	
Complications				
Septic shock	1 (3.6)	0 (0.0)	1 (5.9)	1.000
Persistent candidemia	2 (7.4)	1 (9.1)	1 (5.9)	1.000
Ocular involvement/fundoscopy performed	3/13 (23.1)	1/6 (16.7)	2/7 (28.6)	1.000
Endocarditis/echocardiogram performed	1/15 (6.7)	1/7 (14.3)	0/8 (0.0)	.467
Thrombophlebitis/vascular ultrasound performed	0/2 (0.0)	0 (0.0)	0 (0.0)	-
ICU admission due to fungemia	8 (28.6)	3 (27.3)	5 (29.4)	1.000
Days of ICU stay due to fungemia–median (IQR)	6.00 (4.3–55.8)	5.0 (2–5)	52 (5–77.5)	.134
Days of hospitalization–median (IQR)	31 (22.3–55.3)	27 (22–43)	36 (22–81)	.371
Mortality				
30-day mortality	12 (42.9)	5 (45.5)	7 (41.2)	.823
60-day mortality	12 (42.9)	5 (45.5)	7 (41.2)	.823
1-year mortality	16 (57.1)	8 (72.7)	8 (47.1)	.180
Days from candidemia diagnosis to death—median (IQR)	15.5 (6–37.5)	15 (8.5–182.3)	10 (5.3–26.3)	.189

Catheter-related candidemia was classified according to criterion 1 (positive catheter culture using the Maki technique (15 CFU) or by sonication (1000 CFU).

Abbreviations: CRC, catheter-related candidemia; CVC, central venous catheter; HIV, human immunodeficiency virus; ICU, intensive care unit; IQR, interquartile range; PICC, peripherally inserted central catheter; SD, standard deviation.

*P* values marked in bold indicate significant values (*P* < .05).

Regarding the etiology of species, overall, the most frequently isolated species was *C. albicans* (42.9%), followed in the descending order by *C. parapsilosis* (25.0%), *C. glabrata* (*N. glabrata*) (14.3%), and *C. krusei* (*P. kudriavzevii*) (7.1%), with single cases (3.6% each) of *C. tropicalis*, *C. lusitaniae* (Clavispora lusitaniae), and *C. auris*. According to criterion 1, patients with CRC had a similar proportion to the above-described data, whereas in patients without CRC, *C. parapsilosis* was the most frequent species. Using criterion 2, the trend was similar, with *C. albicans* being the most common species in patients with CRC and *C. parapsilosis* in those without CRC. Primary candidemia accounted for 57.1% of cases, with only 1 case confirmed as having an intra-abdominal origin (3.6%).

When patients with CRC were compared with those without CRC in our cohort, no statistically significant differences were observed in clinical characteristics, comorbidities, hospital setting, risk factors, antifungal treatment, catheter-associated features, or clinical outcomes, except for a higher prevalence of chronic kidney disease in the CRC group (*P* = .050).

The differential time to positivity of BCs, when samples obtained from the catheter were compared with those obtained from peripheral veins, ranged from 12.17 to 12.86 hours according to criteria 1 and 2, respectively, in patients with CRC, and from 12.45 to 12.72 hours in patients without CRC. There were no statistically significant differences between the 2 groups for either criterion (*P* = .91 and *P* = .97, respectively).

The catheter was microbiologically confirmed as the source of candidemia in 39.3% of cases according to criterion 1 and in 53.6% of cases according to criterion 2.

### Microbiological Results According to CRC Criterion 1

Table 2 illustrates the results of the different methods examined for the “conservative” detection of CRC according to criterion 1. The sensitivity of the differential time to positivity of BCs was 72.7%, with a specificity of 52.9%, indicating a considerable proportion of false positives. The PPV was only 50.0%, the NPV was 75.0%, and the overall accuracy was 60.7%.

**Table 2.** Evaluation of Differential Blood Cultures and Fungal Biomarkers for the Diagnosis of Catheter-related Candidemia According to Criterion 1, in Cases From Hospital Gregorio Marañón and Hospital Policlinico San Martino

Test	N	Sensitivity	Specificity	PPV	NPV	Accuracy
Differential time to positivity (≥2 h)	28	72.73% (IC 95%: 45.32%–90.04%)	52.94% (IC 95%: 27.53%–77.00%)	50.00% (IC 95%: 27.28%–72.72%)	75.00% (IC 95%: 46.73%–92.72%)	60.71% (IC 95%: 40.75%–79.43%)
Quantitative differential analysis of BDG (any difference)	28	81.82% (IC 95%: 51.91%–96.23%)	82.35% 82.35% (IC 95%: 56.64%–96.43%)	75.00% (IC 95%: 46.73%–92.72%)	87.50% (IC 95%: 61.76%–97.19%)	82.14% (IC 95%: 63.92%–93.69%)
Quantitative differential analysis of BDG (>20)	28	45.45% (IC 95%: 21.96%–71.55%)	88.24% (IC 95%: 63.05%–97.19%)	71.43% (IC 95%: 35.88%–92.97%)	71.43% (IC 95%: 46.33%–88.04%)	71.43% (IC 95%: 51.62%–87.92%)
Quantitative differential analysis of BDG (>30)	28	36.36% (IC 95%: 14.52%–61.76%)	94.12% (IC 95%: 71.55%–99.24%)	80.00% (IC 95%: 28.45%–99.24%)	65.22% (IC 95%: 40.75%–84.67%)	71.43% (IC 95%: 51.62%–87.92%)
Qualitative differential analysis of CAGTA	28	0.00% (IC 95%: .00%–18.58%)	100.00% (IC 95%: 79.43%–100.00%)	Indeterminate	60.71% (IC 95%: 39.29%–79.43%)	60.71% (IC 95%: 39.29%–79.43%)
Quantitative differential analysis of mannan (any difference)	11	25.00% (IC 95%: 4.35%–63.05%)	42.86% (IC 95%: 12.16%–77.23%)	20.00% (IC 95%: 2.49%–71.55%)	50.00% (IC 95%: 18.34%–81.66%)	36.36% (IC 95%: 10.86%–67.79%)
Qualitative differential analysis of mannan	11	25.00% (IC 95%: 4.35%–63.05%)	85.71% (IC 95%: 48.16%–97.19%)	50.00% (IC 95%: 6.95%–93.05%)	66.67% (IC 95%: 30.70%–90.04%)	63.64% (IC 95%: 30.09%–87.92%)
Quantitative differential analysis of antimannan (any difference)	13	40.00% (IC 95%: 12.16%–73.76%)	25.00% (IC 95%: 3.15%–65.47%)	25.00% (IC 95%: 4.35%–63.05%)	40.00% (IC 95%: 12.16%–73.76%)	30.77% (IC 95%: 9.17%–59.13%)
Qualitative differential analysis of antimannan	13	0.00% (IC 95%: .00%–43.50%)	100.00% (IC 95%: 67.23%–100.00%)	Indeterminate	61.54% (IC 95%: 35.88%–82.75%)	61.54% (IC 95%: 35.88%–82.75%)
Qualitative differential analysis of T2Candida	24	10.00% (IC 95%: 1.67%–41.21%)	78.57% (IC 95%: 49.18%–94.02%)	25.00% (IC 95%: 3.15%–81.49%)	55.00% (IC 95%: 31.17%–77.00%)	50.00% (IC 95%: 30.09%–69.91%)

All values in parentheses represent the 95% confidence interval (CI).

Abbreviations: BDG, 1,3-β-D-glucan; CAGTA, *Candida albicans* germ tube antibodies; NPV, negative predictive value; PPV, positive predictive value.

The qualitative differential analysis of the CAGTA test results revealed no CRC cases, with a sensitivity of 0%.

Differentials of mannan and antimannan biomarkers were investigated both qualitatively and quantitatively. For the mannan antigen, the sensitivity was 25.0%, and the specificity ranged from 42.9% (quantitative determination) to 85.7% (qualitative determination), with a highly variable accuracy (36.4%–63.6%). For the antimannan antibody, the results showed low sensitivity values (0%–40%) and a highly variable specificity (25%–100%).

Regarding the T2Candida PCR, an extremely low sensitivity (10.0%) was found, along with a specificity of 78.6% and a PPV of 25.0%, resulting in an overall accuracy of 50.0%. Moreover, in 2 patients, the results were invalid, and in a third patient, the analysis could not be performed due to insufficient sample volume, which restricts the interpretation of the test.

**Figure 3.**
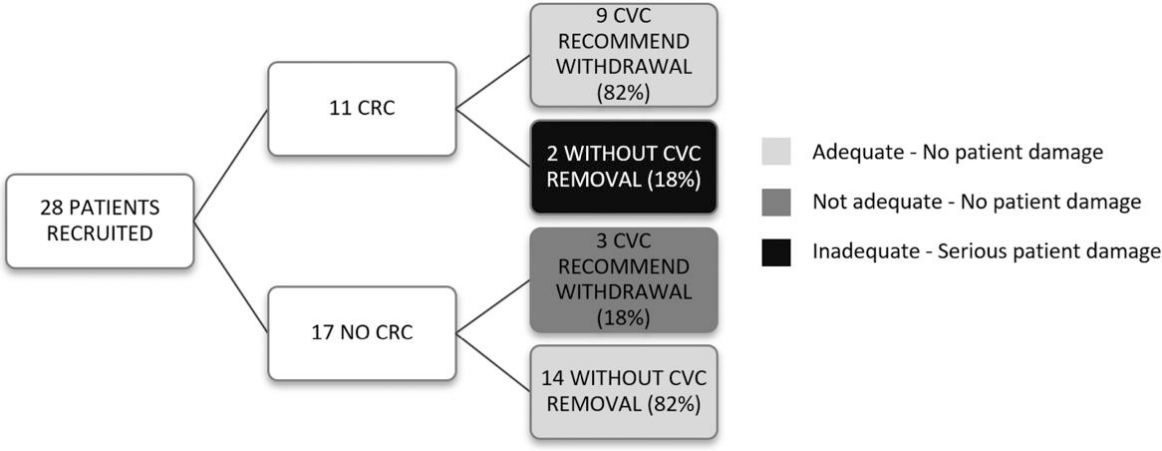
Hypothetical interpretation of 1–3 β-D-glucan (any difference) differential application in patients with candidemia, classified as CRC or non-CRC according to criterion 1. Among patients with confirmed CRC, 82% would be correctly recommended for CVC removal, whereas 18% would be incorrectly advised to retain the CVC, potentially resulting in serious harm. In patients without CRC, 82% of catheters could be safely removed without causing damage, although 18% would hypothetically be retained unnecessarily. CRC, catheter-related candidemia; CVC, central venous catheter.

Quantitative differential analysis of BDG (considering any difference between peripheral and catheter blood as significant) constituted the best diagnostic strategy for the conservative detection of CRC among the investigated tests. The sensitivity was 81.8% (95% confidence interval (CI): 51.9%–96.2%), the specificity was 82.4% (95% CI: 56.6%–96.4%), and the overall accuracy was 82.1% (95% CI: 63.9%–93.7%). Furthermore, the NPV of this biomarker reached 87.50%, the highest among all tests analyzed, and the PPV was 75.0%, the second highest value among all tests evaluated. Nevertheless, when differential thresholds of 20 and 30 pg/mL were applied as BDG criteria, the sensitivity decreased considerably. The mean BDG difference between catheter and peripheral blood in patients with CRC and BDG-positive cases was 71.3 pg/mL according to criterion 1. Figure 3 depicts the hypothetical interpretation of BDG differential use, assuming its application to all patients categorized as CRC or non-CRC based on criterion 1.

### Microbiological Results According to CRC Criterion 2

Table 3 represents the results of the different methods investigated for “conservative” detection, in this case based on the definition of CRC according to criterion 2 (any amount of *Candida* in the catheter culture). Most of the investigated tests demonstrated a reduction in sensitivity, which would significantly restrict their potential clinical applicability. The differential time to positivity of BCs maintained low sensitivity and specificity values of 73.33% and 61.54%, respectively. The quantitative BDG analysis achieved extremely low sensitivities of 25.00%–66.67%, depending on the cutoff applied, with a progressive decrease as the threshold increased. The remaining biomarkers, including mannan, antimannan, and CAGTA, exhibited similarly low and variable sensitivities (0%–50%) and overall accuracies of <55%, thus providing no relevant diagnostic value. Similarly, the T2Candida PCR demonstrated minimal sensitivity (9.09%) and an overall accuracy close to 54%.

**Table 3.** Evaluation of Differential Blood Cultures and Fungal Biomarkers for the Diagnosis of Catheter-related Candidemia According to Criterion 2 in Cases From Hospital Gregorio Marañón and Hospital Policlinico San Martino

Test	N	Sensitivity	Specificity	PPV	NPV	ACCURACY
Differential time to positivity (≥2 h)	28	73.33% (IC 95%: 46.33%–90.04%)	61.54% (IC 95%: 35.88%–82.75%)	68.75% (IC 95%: 41.52%–88.04%)	66.67% (IC 95%: 35.88%–87.36%)	67.86% (IC 95%: 48.16%–84.35%)
Quantitative differential analysis of BDG (any difference)	28	66.67% (IC 95%: 40.06%–86.27%)	84.62% (IC 95%: 57.72%–96.94%)	83.33% (IC 95%: 51.62%–96.43%)	68.75% (IC 95%: 43.14%–87.36%)	75.00% (IC 95%: 55.22%–89.38%)
Quantitative differential analysis of BDG (>20)	28	42.86% (IC 95%: 20.29%–68.52%)	92.86% (IC 95%: 68.19%–98.84%)	85.71% (IC 95%: 48.16%–97.19%)	61.90% (IC 95%: 38.03%–82.75%)	67.86% (IC 95%: 48.16%–84.35%)
Quantitative differential analysis of BDG (>30)	28	25.00% (IC 95%: 9.17%–46.30%)	91.67% (IC 95%: 64.68%–98.42%)	80.00% (IC 95%: 28.45%–99.24%)	47.83% (IC 95%: 27.53%–68.98%)	53.57% (IC 95%: 34.75%–71.55%)
Qualitative differential analysis of CAGTA	28	0.00% (IC 95%: .00%–16.63%)	100.00% (IC 95%: 77.00%–100.00%)	Indeterminate	53.57% (IC 95%: 34.75%–71.55%)	53.57% (IC 95%: 34.75%–71.55%)
Quantitative differential analysis of mannan (any difference)	11	40.00% (IC 95%: 12.16%–73.76%)	50.00% (IC 95%: 18.34%–81.66%)	40.00% (IC 95%: 12.16%–73.76%)	50.00% (IC 95%: 18.34% 81.66%)	45.45% (IC 95%: 17.25%–76.24%)
Qualitative differential analysis of mannan	11	20.00% (IC 95%: 2.49%–71.55%)	83.33% (IC 95%: 43.65%–97.19%)	50.00% (IC 95%: 6.95%–93.05%)	55.56% (IC 95%: 21.96%–84.89%)	54.55% (IC 95%: 23.76%–81.66%)
Quantitative differential analysis of antimannan (any difference)	13	50.00% (IC 95%: 21.05%–78.95%)	28.57% (IC 95%: 3.57%–71.55%)	37.50% (IC 95%: 12.90%–68.98%)	40.00% (IC 95%: 12.16%–73.76%)	38.46% (IC 95%: 15.00%–64.21%)
Qualitative differential analysis of antimannan	13	0.00% (IC 95%: .00%–39.56%)	100.00% (IC 95%: 64.28%–100.00%)	Indeterminate	53.85% (IC 95%: 30.09%–76.24%)	53.85% (IC 95%: 26.24%–79.33%)
Qualitative differential analysis of T2Candida	24	9.09% (<IC 95%: 1.52%–38.03%)	76.92% (IC 95%: 46.33%–94.19%)	25.00% (IC 95%: 3.15%–81.49%)	50.00% (IC 95%: 21.05%–78.95%)	53.85% (IC 95%: 30.09%–76.24%)

All values in parentheses represent the 95% confidence interval (CI).

Abbreviations: BDG, 1,3-β-D-glucan; CAGTA, *Candida albicans* germ tube antibodies; NPV, negative predictive value; PPV, positive predictive value.

## DISCUSSION

Our results indicate that, considering either of the abovementioned diagnostic criteria for CRC, none of the investigated biomarkers, nor the other analyzed techniques (differential time to positivity of BCs or T2Candida), demonstrated sufficient accuracy to exclude a catheter as the source of candidemia without its removal.

Candidemia remains one of the most serious nosocomial infections, with high morbidity and mortality [9, 33]. The majority of patients with candidemia present with a CVC at the time of diagnosis (>85%), which makes it crucial to distinguish CRC from other forms of infection [5, 34]. The literature does not specify the exact percentage of candidemia episodes originating from the catheter, with estimates ranging from 29% to 52% of episodes, depending on the definitions used, hospital records, and microbiological techniques used [7, 35, 36]. Our findings also reflect this variability, wherein using criterion 1, 39.3% of patients were classified as CRC, whereas with the more permissive criterion 2, the proportion increased to 53.6%. These results emphasize the difficulty of establishing a uniform diagnostic criterion to accurately identify CRC episodes.

Catheter removal is considered essential for the prognosis of candidemia episodes [37–39]. In fact, several studies and the recommendations of different guidelines establish source removal as a vital aspect of managing patients with candidemia. This has been recently underscored by the pan-European ECMM Candida III study, which demonstrated a significant survival benefit, showing lower mortality in patients who underwent CVC removal within 24 hours of diagnosis compared to those who did not [40]. Furthermore, a recent analysis from the same dataset revealed that the persistence of a CVC was a major factor significantly associated with persistent candidemia, which in turn leads to increased mortality [41]. Therefore, according to routine clinical practice, systematic catheter removal is adopted [10, 17, 40–42]. This could change if a test with a high NPV was available, which would allow a high proportion of catheters to be preserved in patients with candidemia. According to our results, a proportion of removed catheters were microbiologically sterile, or their cultures remained negative. In this context, Garnacho-Montero et al. previously demonstrated that early catheter removal (within the first 48 hours) was significantly associated with improved survival only in patients suspected of a catheter origin, many of whom were likely catheter-related, whereas catheter management did not influence prognosis in episodes arising from other origins [5]. Nevertheless, although catheter removal remains the current standard of care, our findings suggest that, in selected cases, it could hypothetically be maintained, potentially reducing patient risk and associated healthcare costs when a technical procedure makes it possible to determine with certainty before removal that the catheter is not the cause [43, 44]. Crucially, this approach depends on obtaining results within 24 hours of diagnosis. While traditional batch testing causes unacceptable delays, rapid single-sample platforms like turbidimetric BDG assays render this short turnaround time realistic. Further studies must validate whether this strategy can safely support catheter preservation in clinical practice. Any potential clinical implications are speculative and require validation in larger studies.

Although the differential time to positivity of BCs has been validated in cases of bacteremia, its application in the context of fungal infections has limitations such as longer growth time, and has hence been less investigated, thus indicating that it should not be extrapolated. In our institution, as in other centers, this approach has been dismissed, as previous studies have demonstrated its low specificity for predicting CRC [34, 45–48]. Moreover, our results confirm the futility of classic differential time to positivity Bc methods for diagnosing CRC, as we found low specificity (53%–62%) and PPV (50%–69%) with a high proportion of false positives. Other conservative methods, such as lysis-centrifugation BCs, are time-consuming and have also not demonstrated effectiveness [49, 50].

Currently, some fungal biomarkers such as BDG, CAGTA, mannan, and antimannan are used to guide therapeutic strategies, patient management, and prognosis prediction, as previously documented by our group, which justifies investigating their potential utility for CRC determination [9, 13, 14, 51–53]. Nevertheless, in our cohort, the differentials of the investigated biomarkers exhibited extremely limited diagnostic capacity for the conservative detection of CRC and cannot guide clinical decisions. The CAGTA test did not accurately identify any episodes of CRC, thereby preventing the evaluation of its clinical applicability despite its high specificity. Similarly, tests for mannan and antimannan demonstrated highly variable sensitivities and specificities, thus compromising their utility as diagnostic tools in this context.

Regarding the T2Candida assay, previous studies have demonstrated its usefulness for detecting candidemia faster than BCs, differentiating between complicated and uncomplicated episodes, and identifying patients with poor prognosis [52–$132#?>55]. Nonetheless, the low sensitivity (∼10%) and limited accuracy (∼50%) observed in our study indicate that this technique provides little diagnostic utility for the conservative detection of CRC.

In our study, the quantitative differential analysis of BDG between peripheral and catheter blood samples, considering any quantitative difference in BDG, initially showed the best results among the investigated biomarkers for CRC detection under criterion 1, with sensitivities and specificities of >80%. Nevertheless, we did not consider these values sufficiently robust to establish or exclude a CRC diagnosis. Furthermore, when more restrictive cutoff thresholds (20 or 30 pg/mL) were applied, the sensitivity decreased significantly, thereby limiting its clinical utility.

Moreover, when the more permissive criterion 2 was applied, the sensitivity of BDG decreased drastically (25%–66%), losing almost the entire diagnostic capacity for CRC. These findings emphasize that, although BDG may provide added value in the general diagnosis of invasive fungal infections, it cannot reliably discriminate the catheter as the source of candidemia. Such variability has been reported in studies investigating the overall utility of BDG, but not specifically in the context of CRC diagnosis [56–60], emphasizing that *C. parapsilosis* produces significantly lower levels of BDG than *C. albicans* [61].

The major limitation of this study is the number of recruited patients, which was restricted due to the complexity of the protocol. Hence, the study should be considered consistent with a pilot study design, with limited statistical power to draw definitive conclusions or establish a reliable diagnostic algorithm. Furthermore, in some cases, we excluded patients in whom the CVC had already been removed when BCs turned positive, as well as those in whom the CVC was not withdrawn. This probably resulted in an underestimation of CRC. Moreover, the T2Candida assay does not detect *C. auris*, which may represent a relevant limitation in settings with a higher prevalence of this species, although its impact in our cohort was minimal.

Finally, our overall findings suggest that, despite the risks and costs associated with systematic catheter removal in patients with candidemia, in our opinion, no currently available method is sufficiently reliable to diagnose or exclude CRC before catheter removal. However, despite the limited sample size, which was due to the considerable challenges in patient recruitment, our study provides relevant results that help clarify the limitations of the currently available tests.
